# Translation, validation, and factor structure of the Nepali version of postpartum bonding questionnaires (PBQ-N) among postpartum women in Nepal

**DOI:** 10.1371/journal.pgph.0003469

**Published:** 2024-07-12

**Authors:** Sangita Pudasainee-Kapri, Tumla Shrestha, Thomas Dahan, Mary Wunnenberg

**Affiliations:** 1 School of Nursing-Camden, Rutgers, The State University of New Jersey, New Jersey, United States of America; 2 Maharajgunj Nursing Campus, Institute of Medicine, Tribhuvan University, Kathmandu, Nepal; PLOS: Public Library of Science, UNITED STATES OF AMERICA

## Abstract

This study aimed to translate and test the psychometric properties of the Nepali version of the PBQ (PBQ-N) among postpartum mothers in Kathmandu, Nepal. Data was collected through semi-structured interviews with postpartum mothers (n = 128) of an infant aged one to six months visiting immunization clinics at two selected hospitals in Kathmandu, Nepal. The PBQ scale was translated into Nepali language and backtranslated to English with the help of language and content experts. The PBQ-N was then assessed for factor structure, validity, and reliability. The exploratory factor analysis (EFA) was conducted to examine construct validity of the PBQ-N in which 16 items (*α* = .75) of the original 25 items grouped into three subscales and were found suitable to measure mother-infant bonding among Nepalese women. Regarding convergent validity, a statistically significant, positive correlation was found between the PBQ-N and postpartum depression (*r* = .627, *p* < .001). In addition, a statistically significant, negative association was found between parenting self-efficacy and the PBQ-N (*r* = -.496, *p* < .001). The three subscales demonstrated good internal consistency. Findings indicate adequate estimates of validity and reliability for the PBQ-N in which 16-item measures were considered adequate for screening mother-infant bonding among Nepalese women and are useful for clinical and research purposes. Considering the crucial role of maternal-infant bonding relationships, the use of validated measures is recommended to screen high-risk infants in clinical settings.

## Introduction

Motherhood is a dynamic, life-changing, and ongoing process of transition resulting in changed relationships, routines, assumptions, and roles and responsibilities. An important aspect of the functional transition to motherhood is the bonding process between a mother and their infant [1]. The term, mother-infant bonding, is characterized by the mother’s emotional feelings and affection toward their infant on a biological basis [1, 2]. It also refers to the special bond that exists between mothers and their infants [3]. Positive mother-infant bonding is essential for optimum care, interaction, protection, and well-being of an infant [1, 4]. The bonding relationship between a mother and infant will generally begin during pregnancy and evolve over the first few months of the infant’s life. Bonding that occurs during pregnancy predicts higher postnatal bonding [5, 6]. Failure to establish this bonding relationship during pregnancy may negatively influence mother-infant bonding during the postpartum period [6]. In addition, the emotional bonding and/or attachment relationships established during the early postpartum period may create the foundation for the relationship between mothers and their infants throughout their lives [7]. Thus, early identification of mother-infant bonding difficulties is important to establish healthy mother-infant bonding during the postpartum period [3, 8].

The quality of parenting and maternal mental health status are crucial—in setting a critical foundation for positive mother-infant bonding. Prior research suggests that mother-infant bonding can be affected by maternal self-efficacy or confidence in caring and providing quality time with their children including activities like playing, soothing, caregiving, feeding, and teaching [9]. Furthermore, maternal mental health issues such as maternal depression and anxiety symptoms during pregnancy and or the postpartum period may disrupt the development of healthy maternal-infant bonding patterns [6, 7, 10]. Prior research indicates that maternal depressive symptoms and stress during the second and third trimester of pregnancy were associated with poor mother-infant bonding during the first eight weeks postpartum among an Australian sample [6]. Additionally, mothers who suffered with postpartum depression tend to be less sensitive to their infant’s need for emotional contact and have difficulty in providing essential care such as breastfeeding, interacting with the infant, and maintaining their own hygiene and sleep patterns [7, 11]. Furthermore, research indicates that failure to establish this bond during infancy can have serious long-term effects on the mother-child relationship, thereby negatively affecting the child’s development [1, 2, 5, 12].

Mother-infant bonding is also crucial for a child’s brain development, cognitive and socioemotional development, and their maturational processes [2, 3]. The quality of time that mothers spend with their infant and their bonding relationships has a vital impact on physical, emotional and mental wellbeing of an infant [1]. Poor mother-infant bonding can have a tremendous impact on long-term development. Specifically, mother-infant bonding disorder does not only influence an infant’s socioemotional, behavioral, and cognitive development but is also associated with complications including growth failure, psychosocial disorders, separation anxiety disorders, delinquency and educational problems [3]. Maternal consequences of poor bonding include irritability, hostility, and rejection of the infant. These behaviors may lead to avoidance, neglect, child abuse and maltreatment [4]. Thus, the timely identification of a bonding problem is paramount in minimizing consequences of bonding disruption and long-term developmental outcomes among children.

Postpartum bonding can be assessed by using the Postpartum Bonding Questionnaire (PBQ), a standardized and widely used tool to measure mother-infant bonding The PBQ was originally developed in English in 2001 to screen for bonding disorders among postpartum mothers [12] and was validated among 125 samples in 2005 [13]. The PBQ is a widely used screening tool that can assist in identifying bonding issues between mothers and their infants [13]. It is easy to administer and has good psychometric components with Cronbach’s alphas ranging from 0.64 to 0.95 [1, 8, 12, 13] The PBQ has been validated in many languages including Spanish (3), Flemish [1], Japanese [8, 14, 15], and Tamil [16]. The PBQ is also considered a reliable measure of mother-infant bonding with Cronbach’s alpha in previous studies ranging from 0.64 to 0.95 [8, 12, 13]. However, there is limited research examining mother-infant bonding after childbirth in Nepali context Also, to our knowledge, there is no standardized, translated, and validated instrument available in Nepal to measure mother-infant bonding among postpartum mothers. Thus, the purpose of this study was to translate the standardized English version of the PBQ into Nepali language and to test the psychometric properties (i.e., validity and reliability) of the Nepali version of the PBQ (PBQ-N) among Nepali postpartum mothers of low birth weight (LBW) and normal birth weight (NBW) infants ages one to six months old.

## Materials and methods

### Research design and settings

This is a cross-sectional descriptive multi-site study. A convenient sampling technique was utilized to recruit participants. Research sites include immunization clinics of Kanti Children’s Hospital [KCH] and Tribhuvan University Teaching Hospital [TUTH]) in Kathmandu, Nepal. Postpartum mothers of infants ages one to six months old (n = 128) visiting the immunization clinics of two selected hospitals in Kathmandu were enrolled in this study. Pretesting of instruments was completed among 10 participants before data collection began for this study. Ethical approval from the Nepal Health Research Council and Rutgers University, and institutional review committee approval from the research sites was obtained prior to participant recruitment and data collection. Data collection for this project began on May 15, 2022, after IRB approval and ended in June 2022.

### Ethics statement

The ethical approval for this study was obtained from the Institutional Review Board (IRB) of Rutgers University (IRB #: Pro2022000116), Ethical Review Board (ERB) of Nepal Health Research Council (NHRC) (Reference #: 2799), and the Institutional Review Committee (IRC) of each research sites: Kanti Children’s Hospital and Tribhuvan University Teaching Hospital, prior to begin participant recruitment and data collection.

### Participants and procedure

A total of 128 mothers of infants ages one to six months who visited the immunization clinics of TUTH and KCH for their routine vaccinations were recruited for this study. An equal number of participants from each birth weight category including LBW (n = 64) and NBW (N = 64) groups were enrolled. Postpartum mothers aged 18 to 49 years old who had a singleton birth with an infant one to six months old, who were able to provide infant birth weight and written informed consent met the criteria for inclusion in this study. Any participants under the age of 18 and over 49 years or who were not able to provide the infant’s birth weight, or provide written informed consent were excluded.

All participants provided written informed consent before participating in the study. A semi-structured interview was conducted with the postpartum mothers by research assistants in a private area at the immunization clinics for data collection. The research assistants documented participants’ responses on the standardized questionnaires using a paper-pencil format on the questionnaire sheet. The five-step recommendations for cross-cultural studies [17, 18] were followed as described in the next sections to ensure the language and cultural equivalence, along with pretesting of the instrument prior to data collection.

### Translation and cultural adaptions

Questionnaires were first developed in English and then translated into Nepali for data collection which is the native language of the participants. The five-phase process of establishing cultural equivalence of the measure were followed based on the recommendations of prior research which includes: (a) determining the relevance and function of the phenomena among the study population; (b) translation of the English version of the instruments into Nepali, (c) back-translation into English; (d) testing the translated instruments; and (e) re-evaluation of the process and outcome [17–20]. The EPDS and KPCS measures have been validated and translated into Nepali language [9, 21] and have been used in prior research in Nepal. Language and content experts translated the English version of the PBQ into Nepali and the PBQ-N was then back-translated it into English with the contribution of clinical psychologists from TUTH and KCH and content experts. The TUTH psychologists and research team members of this study reviewed the original translation and back-translation documents carefully and revised the translated document for language and cultural equivalence. Pretesting of the Nepali version of the translated questionnaires was then conducted among 10 participants of both LBW and NBW infants after necessary approvals to ensure the validity and sensitivity of these measures. The PBQ is a publicly available measure for research use, however permission was obtained from the main author of the original English questionnaire to translate it into Nepali language and for use in this study. The ethical approval for this study was obtained from the IRB of Rutgers University, ERB of NHRC, and the IRC of each research sites: Kanti Children’s Hospital and Tribhuvan University Teaching Hospital, prior to participant recruitment and data collection.

### Instruments

#### Sociodemographic characteristics

Data on sociodemographic characteristics including age, ethnicity, educational level, occupation, and family income were collected via participants’ self-reports. Data on infant’s characteristics and anthropometric measures including infant’s age, birth weight (recorded in grams), gestational age at birth, admission to NICU, delivery type, and place of delivery.

#### Perinatal factors

Data on perinatal risk factors included history of maternal mental health issues and or tobacco/alcohol/substance use during pregnancy, history of prior LBW or preterm birth, number of prenatal visits during pregnancy etc.

#### Postpartum depression

Postpartum depression was measured using the Edinburgh Postpartum Depression Scale (EPDS) [22] which consists of 10 items to measure depressive symptoms in the last 7 days with scores ranging from 0 to 30. Participants rated on a 4-point Likert scale (i.e., 0 = never/no, not at all to 3 = yes, very often/yes, most of the time) with higher scores representing increased levels of PPD symptoms. Based on the original authors’ recommendations for referral [22], participants with individual scores of EPDS 10 or greater received a referral to a mental health provider for further evaluation. The items include *“I have been so unhappy that I have had difficulty sleeping*,*” and “I have been anxious or worried for no good reason*.*”* The EPDS has good psychometric properties with Cronbach’s coefficient alpha ranging from 0.74 to 0.87 in prior studies [21, 22]. The validated Nepalese version of the EDPS [21] was used in this study and found to have good internal consistency (α = 0.75).

#### Parenting self-efficacy

The Karitane Parenting Confidence Scale (KPCS) was used to measure participants perceived self-efficacy or confidence in their parenting abilities [23]. The KPCS is a 15-item questionnaire that has been validated among postpartum mothers in Nepal [9]. The KPCS uses a four-point Likert scale (i.e., 0 = hardly ever, 1 = not very often, 2 = some of the time, and 3 = most of the time) with scores ranging from 0 to 45, and higher scores represent greater perceived parenting self-efficacy. Sample items include, *“I am confident about feeding my baby”* and *“I can soothe my baby when he/she is distressed*.*”* The Cronbach’s alpha for the KPCS ranges from 0.81 to 0.87 [9, 23] and test-retest reliability was 0.88 [23]. The Nepali version of the KPCS demonstrates adequate reliability (Cronbach’s alpha coefficient: 0.87) and validity to assess perceived parenting self-efficacy among postpartum mothers [9]. The Cronbach’s coefficient alpha of the KPCS scale in this study was 0.72.

### Mother-infant bonding

The Postpartum Bonding Questionnaire (PBQ) was used to measure mother-infant bonding relationships [12, 13] among mothers one to six months postpartum. The original PBQ has 25 items that consist of four-subscales. Participants rate on a six-point Likert scale (i.e., 0 = never to 5 = always) with scores ranging from 0 to 125 and higher scores representing greater mother-infant bonding impairment [12]. Sample items include, *" I wish my baby would somehow go away”* and “*My baby annoys me*”. The cutoff score of 26 from the total PBQ items would be considered a potential bonding problem that requires further monitoring from the provider and a cutoff score of 40 or higher may suggest a severe bonding disorder [3]. An exploratory factor analysis of PBQ was conducted to determine factor structures, validity, and reliability of the PBQ-N measure. Based on the exploratory factor analysis, 16 items of the PBQ (α = .747) were considered valid and reliable to measure mother-infant bonding among Nepalese samples with details of factor structure, reliability and validity of the scale included in the results section (Refer to the supplementary files for the translated Nepali version of 16-item PBQ and English version of the 16-item PBQ measures).

### Inclusivity in global research

Additional information regarding the ethical, cultural, and scientific considerations specific to inclusivity in global research is included in the Supporting Information.

### Analytic strategy

Data analysis for this research was conducted using Stata MP v18 [24]. First, descriptive statistics were analyzed for the sample. The approach estimating the construct validity of the Nepalese version of the Postpartum Bonding Questionnaire was to perform exploratory factor analysis using principal components factors. Parallel analysis, a data driven strategy to identify the number of factors relative to a simulated random data set [25], was used to reduce the 25 items to a set of scales with an adjusted eigenvalue of one selected as the cut-off for retention in the factor model. The data was then subjected to orthogonal rotation to facilitate interpretation of the scales present in the data [26]. After identifying the underlying factor structure, Cronbach’s coefficient alpha was calculated for each scale to determine its internal consistency reliability based on the recommendations of prior study [27].

Convergent validity, the subtype of criterion validity, was estimated by Spearman’s Rank Correlation Coefficient with two intact scales: the Karitane Parenting Confidence Scale and the Edinburgh Postnatal Depression Scale. The selection of the rank correlations relaxes normality assumptions of traditional Pearson’s Product Moment Correlations by instead converting scores to ranks [28]. The KPCS has been translated in Nepali language and validated for use with Nepali postpartum mothers [9]. This measure was originally validated to use for mothers of children up to 12 months of age and has good convergent validity with EPDS [23]. The EPDS is also a translated and validated tool among Nepalese postpartum mothers [21] and has been used to measure postpartum depression among Nepalese sample [29]. These scales were selected to measure correlation with Nepali version of the PBQ as they measure some similar constructs obtained at the same time and have been used in other translation validation (i.e., KPCS and EPDS) [23] and PBQ validation studies [8, 14], making them good candidate measures for validation purposes, a strategy employed in other translations of the PBQ (30). These scales were selected because they each measure a similar construct to mother-infant bonding but do not directly measure that construct, making them good candidate measures for validation purposes, a strategy employed in other translations of the PBQ [30]. Any subscales that failed to converge with the other measures were ultimately rejected as a candidate measure for the final version of the scale.

After identifying the underlying factor structure and establishing convergent validity between measures, Cronbach’s coefficient alpha was calculated for each subscale to determine its internal consistency reliability [27]. Items were then summed to create a final version of the overall scale measure.

## Results

### Sample demographic information of participants

Table 1 contains information about the sample’s demographic characteristics. The sample of 128 women were between one to six months postpartum, with the most responses recorded approximately 2 months after giving birth. The mean age of infants was 2.75 months (*SD* = 1.29), and the mean age of postpartum women was 27.99 years (*SD* = 4.28). More than half participants had a cesarean birth (58.59%) followed by normal vaginal delivery (39.84%). Participants were ethnically diverse with majority of them were Brahmin and Chhetri (50%), and Janajati (34.4%) and least were Madhesi (7.8%), Dalit (7%), and Muslim (0.8%). 39% of sample of mothers who participated in this study had a Bachelor’s degree or higher level of education with 40% of fathers having completed a bachelor’s degree or higher.

**Table 1:** Demographic characteristics of study participants (N = 128).

Variables	N/Mean (% / SD)
Infant age in months	2.750 (1.286)
Infant sex	
Female	67 (52.3%)
Male	61 (47.7%)
Participant’s ethnicity	
Dalit	9 (7.0%)
Janajati	44 (34.4%)
Madhesi	10 (7.8%)
Muslim	1 (0.8%)
Brahmin and Chhetri	64 (50.0%)
Place of infant’s birth	
Home	2 (1.6%)
Government hospital/Public health center	112 (87.5%)
Private Hospital	14 (10.9%)
Type of delivery	
Vaginal	51 (39.8%)
Vaginal Assisted Delivery	2 (1.6%)
Caesarean Section	75 (58.6%)
Participant (Mother) education Unable to read and write Primary Secondary Higher Secondary Bachelor Master’s level/higher Fathers’ education Unable to read and write Primary Secondary Higher Secondary Bachelor Master’s level/higher	3 (2.3) 3 (2.3) 30 (23.4) 42 (32.8) 25 (19.5) 25 (19.5) 2 (1.6) 4 (3.1) 35 (27.3) 36 (28.1) 24 (18.8) 27 (21.1)
Income sufficient for household expenditure	
<6 months	7 (5.5%)
6–11 months	15 (11.7%)
12 months	66 (51.6%)
>12 months	40 (31.2%)

#### Factor structure of the postpartum bonding questionnaire

An EFA using parallel analysis of the 25 PBQ items yielded a four-factor structure, with adjusted eigenvalues for the first four components greater than 1. The rotated factor loadings are presented in Table 2. The first factor related to the *Lack of Affection Towards the Baby* loaded on six items with factor loadings ranging from .41 to .67. These items together included those that were reverse coded and were associated with a lack of affection towards the baby. The second factor also loaded on six items, with factor loadings ranging from .35 to .65. These items are related to *Maternal Anxiety and Frustration*. The third factor related to *Regretfulness About Having the Baby* consisted of 4 items with loadings ranging from .46 to .63. Finally, the fourth factor contained two items related to *Confidence in Caring for the Baby*, with high factor loadings of .77 and .71. Among the 25 original items, three items were dropped from the analysis because of no variation in scores for the items reflecting resentful and harm/hurtful actions towards the baby (sample items include: “*I resent my baby”*, *“I have done harmful things to my baby”*, and “*I wish my baby would somehow go away*.*”* Four other items had more than 90% variance in their scores unexplained by the common factors and were not retained for final analysis.

**Table 2:** Results of factor analysis.

	Factor1	Factor2	Factor3	Factor4
Adjusted Eigenvalue^a^ (% Variance Explained by Factor^b^)	2.83 (15.1)	1.70 (15.0)	1.39 (11.4)	1.15 (10.1)
My baby cries too much	**0.74**	0.02	0.00	-0.04
My baby makes me feel anxious	**0.73**	0.12	-0.02	-0.04
My baby annoys me	**0.67**	0.08	0.33	-0.15
My baby irritates me	**0.50**	0.17	0.18	0.32
I feel angry with my baby	**0.47**	0.05	0.02	-0.06
I am afraid of my baby	**0.45**	0.13	-0.05	0.38
I feel happy when my baby smiles or laughs	-0.05	**0.75**	0.29	0.04
I think my baby is the most beautiful baby in the world	0.24	**0.73**	0.06	0.02
I enjoy playing with my baby	0.17	**0.71**	-0.04	-0.12
I love my baby to bits	-0.10	**0.71**	-0.10	0.02
I love to cuddle my baby	0.16	**0.51**	-0.17	-0.08
I feel close to my baby	0.03	**0.51**	-0.15	-0.07
I regret having this baby	-0.24	0.03	**0.77**	-0.02
I feel trapped as a mother	0.31	0.00	**0.72**	-0.03
My baby winds me up	0.41	0.02	**0.59**	-0.15
I wish the old days when I had no baby would come back	0.19	0.08	**0.53**	0.05
I feel confident when caring for my baby	0.00	0.05	-0.05	**0.88**
I feel the only solution is for someone else to look after my baby	-0.10	-0.12	-0.01	**0.84**

*Notes*: a. Bias-adjusted eigenvalue from parallel analysis before rotation. b. Percentage of variance explained by principal components factors after orthogonal varimax rotation.

#### Correlations with other measures

To determine the convergent validity of the instrument, the subtype of criterion validity, the subscales and overall scale were compared to the EPDS and the KPCS. The Spearman’s Rank Correlation Coefficients are presented in Table 3. The results indicate that the overall scale has evidence of convergent validity with the EPDS and with the KPCS. Furthermore, the subscales for *Anxiety*, *Affection*, and *Regret* all feature these same characteristics.

**Table 3:** Pairwise correlations between measures.

	1	2	3	4	5	6	7
1. Final PBQ- N 16	-						
2. Affection	0.487***	-					
3. Anxiety	0.916***	0.297***	-				
4. Regret	0.615***	0.073	0.434***	-			
5. Confidence (Rejected)	0.116	0.030	0.132	0.025	-		
6. PBQ-N18 Scale (Rejected)	0.888***	0.383***	0.839***	0.532***	0.471***	-	
7. KPCS	-0.496***	-0.229**	-0.470***	-0.274**	-0.138	-0.425***	-
8. EPDS	0.627***	0.357***	0.583***	0.340***	-0.068	0.512***	-0.408***

*** p < .001

** p < .01

* p < .05.

Note. KPCS: Karitane Parenting Confidence Scale; EPDS: Edinburgh Postpartum Depression Scale

The *Confidence* subscale is not correlated with the KPCS or EPDS or any of the other subscales of the PBQ and has only a modest correlation with the overall PBQ measure (*ρ* < .05). For this reason, this scale was not included in the final version of the PBQ-N. We also calculated a version of the overall scale with 18 items (including the rejected *Confidence* subscale), and it similarly underperformed relative to the final 16-item version in terms of convergent validity with both the EPDS and the KPCS, further justifying the exclusion of these items from the final measure.

#### Scale reliability of the PBQ scale and subscales

Using Cronbach’s Alpha Coefficients, we assessed each factor independently to determine if the items reliably measured the underlying construct and together to determine if the overall scale was a reliable instrument. The results are presented in Table 4. Coefficient Alpha for the *Regretfulness About Having the Baby* subscale was lower than desirable (.559), while the other scales meet Nunnally’s minimally acceptable threshold of .7 [31]. This is also consistent with the recommendation of Heale and Twycross who indicated the reliability score of .7 or higher is acceptable [32]. The overall scale reliability of PBQ-N in this study was .747, which DeVellis considers respectable [27]. The rejected 18-item version underperformed in terms of internal consistency with a Cronbach’s Alpha of .694. Thus, the total combined PBQ-N score of 16 items ranges from 0 to 80 with higher scores indicates increased mother-infant bonding disorder.

**Table 4:** Descriptive statistics and internal reliability of scales (N = 128).

	M	SD	Skew	Kurt	Coef Alpha
I feel happy when my baby smiles or laughs	0.086	0.281	2.955	9.730	
I think my baby is the most beautiful baby in the world	0.141	0.430	3.159	12.389	
I enjoy playing with my baby	0.211	0.512	2.750	11.359	
I love my baby to bits	0.055	0.228	3.917	16.344	
I love to cuddle my baby	0.250	0.575	3.180	16.925	
I feel close to my baby	0.156	0.386	2.299	7.453	
**Affection Subscale (total)**	**0.898**	**1.616**	**2.368**	**8.389**	** 0.714**
My baby makes me feel anxious	0.820	0.967	0.627	2.133	
My baby cries too much	1.273	0.978	0.294	2.932	
My baby annoys me	0.641	0.839	0.834	2.178	
My baby irritates me	0.945	0.975	0.571	2.547	
I feel angry with my baby	0.320	0.651	1.804	4.752	
I am afraid of my baby	0.109	0.473	5.697	40.822	
**Anxiety and Frustration Subscale (total)**	**4.109**	**3.163**	**0.752**	**3.524**	** 0.697**
I feel trapped as a mother	0.313	0.718	2.405	8.758	
I regret having this baby	0.016	0.125	7.811	62.016	
My baby winds me up	0.523	0.947	1.747	5.473	
I wish the old days when I had no baby would come back	0.227	0.578	2.402	7.282	
**Regret Subscale (total)**	**1.078**	**1.742**	**2.304**	**10.671**	** 0.559**
**Nepal PBQ Total Score (16 items)**	**6.086**	**4.838**	**1.008**	**4.512**	** 0.747**

## Discussion

### Key findings

The current study demonstrated the adequate validity and reliability of a 16-item PBQ-N among Nepali postpartum mothers during one to six months postpartum. The 16-item measure of the PBQ-N is suitable to measure mother-infant bonding issues among postpartum mothers of LBW (both pre-term and full term LBW) and NBW infants. An EFA using parallel analysis of the 25-item original English version of the PBQ yielded four interrelated factors with 18 items. Due to the lack of correlations of the fourth factor, the *Confidence Subscale* of the PBQ-N, with the EPDS, the KPCS, and other subscales of the PBQ-N, the two items of the *Lack of Confidence in Caring for the Baby* subscale were removed from the final version of the PBQ-N. Thus, after further analysis of the PBQ-N, this study demonstrated that three factor structure of the PBQ-N including *Lack of Affection Towards the Baby (6 items)*, *Maternal Anxiety and Frustration (6 items)*, *and Regretfulness About Having the Baby (4 items)*, represented with good reliability and validity.

Consistent with the definition of mother-infant bonding, each sub-scale is aligned to different aspects of maternal emotions and behaviors towards their infants. The original English version of the PBQ has four factor structure including *Impaired Bonding*, *Rejection and Pathological Anger*, *Anxiety about Care of the Baby*, *and Risk of Incipient Abuse* [12, 13]. Although the findings with a three-factor model confirmed in this study support the original PBQ validation study among samples from England [12, 13] and a validation study among Japanese sample (14), the factor structure or items loadings on each of the factor did not always support the original validation study findings. For example, the *Lack of affection* subscale in this data consisted of the same six items as found in the Japanese version [14]. This finding offers strong confirmatory evidence of that factor because “the likelihood those results being a quirk is quite small” [30] across two different samples in two different languages. Other previous studies also reported some variations on factor structure of the PBQ including a three-factor structure in Japanese [8] and Tamil sample [16], and a one-factor structure in a Flemish sample [1] Moreover, from the 25 original PBQ items, the EFA model shows reasonable fit of the data with 16 items of the Nepali version of the PBQ. Consistent with the results of this study, prior validation studies also reported some variations in the number of items that fit with the model ranging from 14–21 items [1, 14, 16].

Contrary to the initial PBQ validation [12, 13], the *Resentfulness/Incipient Abuse* related factor was found to be not relevant in the Nepalese sample. In particular, this study did not find any variation in *Resentfulness/Incipient Abuse* related questions among a Nepalese sample, thus three items measuring incipient abuse or resentfulness were dropped from the final analysis. In the original PBQ study, the incipient abuse related factor only accounted for 3.4% of the total variance [12]. Although some studies recommend excluding these items because of their poor validity, we included all items of the original PBQ in this sample to identify its relevance in a Nepalese sample and or Nepalese cultural contexts. In addition, four other items were dropped due to having more than 90% variance in their scores that were unexplained by the common factors. The fourth factor in the present study, *Lack of Confidence in Caring for the Baby*, had the strongest factor loadings across all four factors, however, its lack of correlation with the overall PBQ scale measure and lack of convergent validity with the KPCS and EPDS was concerning, and this subscale was ultimately not retained. This resulted in a total of 16 items from the original 25. The possible reason for the differences in factor structure and/or the total items in the PBQ scale in different validation studies might be due to the population characteristics [1] and or cultural factors. In particular, this study recruited participants from the immunization clinics of tertiary hospitals in Kathmandu whereas other studies recruited samples from hospitals [14] and or community settings [1]. The social and cultural values related to the maternal role in caring for an infant may contribute in positive mother-infant bonding relationships [8, 14]. The difference in factor structure with the original versions of the English and PBQ-N could be further explained by cultural differences in expectations of mothers’ role in caring an infant between two countries as evidenced in other validation studies with similar contexts [16]. In addition, participants in this study were interviewed by research assistants who asked the participants responses to each of the survey questions for cultural and language related adaptation needs rather than participants completing the questionnaires independently as in the original validation study. The interview method for data collection was conducted to increase diversity and inclusivity of participants from diverse ethnic backgrounds and those with a lower educational level who generally are unable to read, comprehend, and complete those questionnaires. Thus, participants in this study may have not provided accurate information/responses to questions regarding their bonding relationships with their infant due to stigma impacting their self-reported responses. Also, the expression of anger and or incipient abuse are viewed seriously in Nepal and not culturally accepted consistent to other contexts, like India [16].

Moreover, the findings of this study highlight that the three-factor structure of the PBQ-N is considered a reliable and valid tool to measure mother-infant bonding in Nepali postpartum women with Cronbach’s alpha coefficients ranging from 0.559 to 0.714. Although the Cronbach’s alpha for the *Regretfullness About Having the Baby* subscale in this study was lower than desirable (.559), the Cronbach alpha scores for other sub-scales were near or above .7 and the overall PBQ-N scale Cronbach’s alpha was 0.747 that demonstrates good reliability of PBQ-N as recommended by prior studies [27, 31, 32]. These findings are similar with prior studies which demonstrated good reliability of PBQ with Cronbach’s coefficient alphas ranging from 0.64 to 0.82 [8] and 0.887 [1].

Predictions regarding the convergent validity of the PBQ were also confirmed in that the subscales of the PBQ-N and the overall scale were compared to the EPDS and KPCS. All sub-scales of the PBQ-N and the total scale were correlated with the KPCS except for the *Lack of Confidence in Caring for the Baby* subscale which demonstrates good convergent validity of this measure. Similar to prior study findings [8, 14, 30], this study also indicated a positive association between postpartum depressive symptoms and mother-infant bonding which shows good convergent validity. The subscales of the PBQ-N; *Anxiety*, *Affection*, *and Regret* subscales were also moderately correlated with the EPDS scale. These findings are supported by the Japanese PBQ validation study of a 14-item version of the PBQ [14]. In that study, the EPDS score was significantly correlated with the total PBQ-14 scale as well as with the subscales including *‘Impaired Bonding’*, moderately correlated with ‘*Anxiety About Care*’, and slightly correlated with *‘Rejection and Anger’* and *‘Lack of Affection’*]. Another validation study in a Japanese sample also indicated significant associations among the EPDS with each of the PBQ subscales in mothers one month postpartum [8]. Another study among Japanese mothers reported correlations of the EPDS with the subscales; *Lack of Affection and Anger and Rejection* indicating the relationship between the psychopathology of depressive symptoms and mother-infant bonding [15]. Along with validation studies, the findings of this study were consistent with prior research demonstrating the positive significant association between EPDS and mother-infant bonding among Tamil [16], Japanese [14, 33], and Ethiopian [34] sample.

The major strength of this study is that although the PBQ is a commonly used tool to identify mother-infant bonding disorders among postpartum women and has been validated in many languages including Spanish [3], Flemish [1], Japanese [8, 14, 30], and Tamil [16] to detect mother-infant bonding problems, this is the first validation study among a Nepalese sample of postpartum mothers. Another strength of this study is the inclusion of participants from diverse ethnic groups and educational levels with infants born normal birth weight and low birth weights and/or preterm birth from immunization clinics. In addition, this study reported on the translation, validation, and cultural adaptation of the PBQ-N to measure mother-infant bonding in the context of Nepal, a lower-middle income country that can be adapted in similar contexts. It also addresses an existing gap, as to date there are very limited options for evaluating mother-infant bonding in the context of Nepal. Thus, the PBQ-N tool is a valid and reliable instrument for use in clinical settings and for research purposes among Nepali postpartum mothers of infants up to six months of age.

### Limitations

Although this is the first study to translate and validate the PBQ in Nepali language, there are some notable limitations of the study. The PBQ was originally validated as a self-administered tool, but for this study data from participants was obtained through semi-structured interviews and participants responses to standardized questionnaires were recorded in the paper-pencil format by the research assistants to capture diverse participants with lower levels of education and those who were unable to read and write. Therefore, the information provided by participants about their bonding might be less accurate due to the stigma associated with self-reports of bonding disorders. Due to the data limitations in the cross-sectional study, the researchers were not able to calculate the test-retest reliability of the study measure. In addition, only two measures were used in the validation of the PBQ-N including, EPDS and KPCS, and that future research would need to extend the range of validation measures, specifically, to include measures that have closer conceptual associations with the identified sub-scales of the Nepali PBQ version, and to assess other aspects of validity than convergent validity. Lastly, the generalizability of the findings is limited across all postpartum mothers of an infant in Nepal due to the select sample of postpartum mothers from two selected tertiary hospitals in Kathmandu, Nepal. But it is noteworthy that the postpartum women in this study were not only permanent residents of the Kathmandu district but there were also participants from other diverse geographic locations across the country and those residing temporarily in the capital city for their work and business purposes.

### Conclusion and implications

Findings of the present study indicate that the Nepali version of the PBQ is a valid and reliable instrument for measuring postpartum bonding in Nepalese mothers of LBW and NBW infants up to six months postpartum. Further analysis indicated that the 16-item PBQ-N is considered adequate for clinical and research purposes among Nepalese samples. Specifically, the 16 items of the PBQ-N with a three-factor model including *Lack of Affection Towards the Baby* (6 items), *Maternal Anxiety and Frustration* (6 items), and *Regretfulness About Having the Baby* (4 items) is valid and reliable for detecting mother-infant bonding issues among Nepalese postpartum mothers. Considering the crucial role of mother infant bonding relationships for infant development, the standardized, translated, and validated measures of the PBQ-N is recommended for use in clinical settings. The PBQ-N can be utilized to screen high-risk mothers of infants and to promote timely identification, and management of postpartum bonding disorders in Nepalese mothers during critical stages of infant development.

The lack of a validated and reliable instrument may contribute to the lack of a standardized practice in clinical settings for screening Nepalese mothers for issues with maternal-infant bonding. The translation and validation of the PBQ-N has important implications for primary care providers, pediatricians, and other clinicians to detect postpartum bonding disorders early and facilitate timely referral for appropriate management. Findings from studies that utilize the Nepali version of the PBQ can provide data to assist in the development of multilevel interventions for mothers and infants with a bonding disorder. It can also lead to practice changes to include the use of the PBQ-N as a routine screening during visits to the immunization clinics and pediatric primary care practices. Future studies should validate the Nepali version of the PBQ in a larger sample using self-reported questionnaires from mothers during the first year after birth. Early identification of mother-infant bonding issues is imperative to prevent future short-and long-term developmental consequences among at-risk infants. Thus, future studies should also identify diagnostic findings and clinical indicators of maternal-infant bonding disorders to measure the sensitivity and specificity to this scale. In addition, although we use the interview method to collect responses of participants on the PBQ questionnaire in this study, additional study should be conducted to measure PBQ using self-reported methods among Nepalese postpartum mothers and compare those results to identify if interview and self-reports are two equivalent forms of measurement among this population.

## Supporting information

S1 Data Aggregated data files. (XLSX)

S2 Data Data summary and results- summary statistics document with details. (LOG)

S1 Text Nepali version of the postpartum bonding questionnaire (PBQ-N)-16 items. (DOCX)

S2 Text English version of the postpartum bonding questionnaire (PBQ)-16 items. (DOCX)

S3 Text PLOS’ questionnaire on inclusivity in global research. (DOCX)

## References

[1] Roxanne B, Laura VdB, Yannic vG, Natacha VdC, Luka VL, Kuipers YJ Validation of the postpartum bonding questionnaire: A cross-sectional study among Flemish mothers. Midwifery. 2022;107:103280–103288. doi: 10.1016/j.midw.2022.103280 35182820

[2] Farré-Sender B, Torres A, Gelabert E, Andrés S, Roca A, Lasheras G, et al. Mother–infant bonding in the postpartum period: assessment of the impact of pre-delivery factors in a clinical sample. Archives of women’s mental health. 2018;21(3):287–297. doi: 10.1007/s00737-017-0785-y 29046965

[3] Garcia-Esteve L, Torres A, Lasheras G, Palacios-Hernández B, Farré-Sender B, Subirà S, et al. Assessment of psychometric properties of the Postpartum Bonding Questionnaire (PBQ) in Spanish mothers. Archives of women’s mental health. 2015;19(2):385–94. doi: 10.1007/s00737-015-0589-x 26608303

[4] Bicking Kinsey C, Hupcey JE. State of the science of maternal–infant bonding: A principle-based concept analysis. Midwifery. 2013;29(12):1314–1320. 10.1016/j.midw.2012.12.019 23452661 PMC3838467

[5] Le Bas GA, Youssef GJ, Macdonald JA, Rossen L, Teague SJ, Kothe EJ, et al. The role of antenatal and postnatal maternal bonding in infant development: A systematic review and meta‐analysis. Social development (Oxford, England). 2020;29(1):3–20.

[6] Rossen L, Hutchinson D, Wilson J, Burns L, A Olsson C, Allsop S, et al. Predictors of postnatal mother-infant bonding: the role of antenatal bonding, maternal substance use and mental health. Archives of women’s mental health. 2016;19(4):609–622. doi: 10.1007/s00737-016-0602-z 26867547

[7] Glasheen C, Richardson GA, Kim KH, Larkby CA, Swartz HA, Day NL. Exposure to maternal pre- and postnatal depression and anxiety symptoms: Risk for major depression, anxiety disorders, and conduct disorder in adolescent offspring. Development and psychopathology. 2013;25(4pt1):1045–63. doi: 10.1017/S0954579413000369 24229548 PMC4310683

[8] Ohashi Y, Kitamura T, Sakanashi K, Tanaka T. Postpartum Bonding Disorder: Factor Structure, Validity, Reliability and a Model Comparison of the Postnatal Bonding Questionnaire in Japanese Mothers of Infants. Healthcare (Basel). 2016;4(3):50–61. doi: 10.3390/healthcare4030050 27490583 PMC5041051

[9] Shrestha S, Adachi K, Shrestha S. Translation and validation of the Karitane Parenting Confidence Scale in Nepali language. Midwifery. 2016;36:86–91. doi: 10.1016/j.midw.2016.03.004 27106948

[10] Parfitt Y, Ayers S. Transition to parenthood and mental health in first‐time parents. Infant Mental Health Journal. 2014;35(3):263–73. doi: 10.1002/imhj.21443 25798480

[11] Muzik M, Bocknek EL, Broderick A, Richardson P, Rosenblum KL, Thelen K, et al. Mother–infant bonding impairment across the first 6 months postpartum: the primacy of psychopathology in women with childhood abuse and neglect histories. Archives of women’s mental health. 2013;16(1):29–38. 10.1007/s00737-012-0312-0 PMC4040083 23064898

[12] Brockington IF, Oates J, George S, Turner D, Vostanis P, Sullivan M, et al. A Screening Questionnaire for mother-infant bonding disorders. Archives of Women’s Mental Health. 2001;3(4):133–40.

[13] Brockington IF, Fraser C, Wilson D. The Postpartum Bonding Questionnaire: a validation. Archieves of Womens Mental Health. 2006;9(5):233–42. doi: 10.1007/s00737-006-0132-1 16673041

[14] Suetsugu Y, Honjo S, Ikeda M, Kamibeppu K. The Japanese version of the Postpartum Bonding Questionnaire: Examination of the reliability, validity, and scale structure. Journal of psychosomatic research. 2015;79(1):55–61. doi: 10.1016/j.jpsychores.2015.02.008 25818345

[15] Ohara M, Okada T, Kubota C, Nakamura Y, Shiino T, Aleksic B, et al. Validation and factor analysis of mother-infant bonding questionnaire in pregnant and postpartum women in Japan. BMC psychiatry. 2016;16(1):212–219. doi: 10.1186/s12888-016-0933-3 27389341 PMC4936305

[16] Vengadavaradan A, Bharadwaj B, Sathynarayanan G, Durairaj J, Rajaa S. Translation, validation and factor structure of the Tamil version of the Postpartum Bonding Questionnaire (PBQ-T). Asian J Psychiatr. 2019;40:62–7. doi: 10.1016/j.ajp.2019.01.018 30739859

[17] Jones PS, Lee JW, Phillips LR, Zhang XE, Jaceldo KB. An Adaptation of Brislin’s Translation Model for Cross-cultural Research. Nursing Research (New York). 2001;50(5):300–4. doi: 10.1097/00006199-200109000-00008 11570715

[18] Brislin RW. Back-Translation for Cross-Cultural Research. Journal of Cross-Cultural Psychology. 1970;1(3):185–216. 10.1177/13591045700010030

[19] Berry JW. On Cross-Cultural Comparibility. International Journal of Psychology. 1969;4(2):119–28.

[20] Phillips LR, De Hernandez IL, Torres De Ardon E. Strategies for achieving cultural equivalence. Research in Nursing & Health. 1994;17(2):149–54. doi: 10.1002/nur.4770170210 8127995

[21] Bhusal BR, Bhandari N, Chapagai M, Gavidia T. Validating the Edinburgh Postnatal Depression Scale as a screening tool for postpartum depression in Kathmandu, Nepal. International Journal of Mental Health Systems. 2016;10(1):71–78. doi: 10.1186/s13033-016-0102-6 27785152 PMC5073833

[22] Cox JL, Holden JM, Sagovsky R. Detection of postnatal depression. Development of the 10-item Edinburgh Postnatal Depression Scale. British Journal of Psychiatry. 1987;150(6):782–6. doi: 10.1192/bjp.150.6.782 3651732

[23] Črnčec R, Barnett B, Matthey S. Development of an instrument to assess perceived self-efficacy in the parents of infants. Research in Nursing & Health. 2008;31(5):442–53. doi: 10.1002/nur.20271 18297638

[24] StataCorp. Stata MP v18. Published online 2021.

[25] Dinno A. Implementing Horn’s parallel analysis for principal component analysis and factor analysis. The Stata Journal. 2009;9(2):291–298. 10.1177/1536867X0900900

[26] Abdi H. Factor rotations in factor analyses. In Lewis-Beck M. Bryman A., & Futing T(Eds.), Encyclopedia for Research Methods for the Social Sciences.: Sage; 2003.

[27] DeVellis RF. Scale Development: Theory and Applications: SAGE Publications; 2012.

[28] Howell DC. Fundamental statistics for the behavioral sciences. Cengage; 2010.

[29] Singh DR, Sunuwar DR, Adhikari S, Singh S, Karki K. Determining factors for the prevalence of depressive symptoms among postpartum mothers in lowland region in southern Nepal. PLoS One. 2021, 22;16(1): e0245199. doi: 10.1371/journal.pone.0245199 33481863 PMC7822291

[30] Kaneko H, Honjo S. The psychometric properties and factor structure of the postpartum bonding questionnaire in Japanese mothers. Psychology. 2014;5;1135–1142.

[31] Nunnally JC. Psychometric Theory. McGraw-Hill; 1978.

[32] Heale R, & Twycross A. Validity and reliability in quantitative studies. Evidence-Based Nursing. 2015, 18(3), 66–67. doi: 10.1136/eb-2015-102129 25979629

[33] Nakano M, Upadhyaya S, Chudal R, Skokauskas N, Luntamo T, Sourander A, et al. Risk factors for impaired maternal bonding when infants are 3 months old: A longitudinal population based study from Japan. BMC Psychiatry. 2019; 19:87–96. 10.1186/s12888-019-2068-9 30849963 PMC6408752

[34] Hailemeskel HS, Kebede AB, Dagnaw FT. Mother-infant bonding and its associated factors among mothers in the postpartum period, Northwest Ethiopia, 2021. Frontiers in Psychiatry. 2022; 13; 13:893505. doi: 10.3389/fpsyt.2022.893505 35911218 PMC9326158

