# Supplementary material for: Translation, validation, and factor structure of the Nepali version of postpartum bonding questionnaires (PBQ-N) among postpartum women in Nepal
Source: PLOS Glob Public Health. 2024 Jul 12;4(7):e0003469. doi: 10.1371/journal.pgph.0003469 (PMC11244797; doi:10.1371/journal.pgph.0003469)
Supplement: S1 Text — (DOCX) [file pgph.0003469.s003.docx]

**jRrf hGd]kl5sf] cfdf–aRrf larsf] cfTdLo ;DjGw dfkg ug]{ k|ZgfjnL**

tnsf jfSof+zx? tkfO{ nfO{ sltsf] l7s nfU5g\, s[kof eGg'xf];\ . tkfO{n] lbPsf] pQ/ cfk+m}df 7Ls jf j]7Ls eGg] x'+b}g . cfkm\gf] xfn;fnsf] cg'ejsf] cfwf/df ;xL pQ/ 5fGg'xf];\ .

| **qm=;+=** | **jfSof+zx?** | **pQ/sf] nflu lasNkx?** | | | | | |
| --- | --- | --- | --- | --- | --- | --- | --- |
| != | d d]/f] jRrf;+u glhs ePsf] dxz'; u5'{ . | ;w}e/L | w]/} h;f] | k\|fo h:ff] | slxn]sf+xL | la/n} | slxNo} klg |
| * @= | d d]/f] jRrf gx'b}sf ;do kmls{pg eGg] rfxG5' . | ;w}e/L | w]/} h;f] | k\|fo h:ff] | slxn]sf+xL | la/n} | slxNo} klg |
| #= | d d]/f] jRrfnfO{ sfvdf lng jf c+ufnf] xfNg dgk/fp5' . | ;w}e/L | w]/} h;f] | k\|fo h:ff] | slxn]sf+xL | la/n} | slxNo} klg |
| * $= | dnfO{ of] jRrf ePsf]df k5'tf] 5 . | ;w}e/L | w]/} h;f] | k\|fo h:ff] | slxn]sf+xL | la/n} | slxNo} klg |
| * %= | dnfO{ d]/f] jRrfn] cNemfO{/x]sf] h:tf] dx;'; x'G5 . | ;w}e/L | w]/} h;f] | k\|fo h:ff] | slxn]sf+xL | la/n} | slxNo} klg |
| ^= | d d]/f] jRrfnfO{ w]/} dfof ub{5' . | ;w}e/L | w]/} h;f] | k\|fo h:ff] | slxn]sf+xL | la/n} | slxNo} klg |
| &= | d]/f] jRrf xf:bf jf d':s'/fpbf dnfO{ w]/} v'zL nfU5 . | ;w}e/L | w]/} h;f] | k\|fo h:ff] | slxn]sf+xL | la/n} | slxNo} klg |
| * * | dnfO{ d]/f] aRrfn] l/; p7fpF5 . | ;w}e/L | w]/} h;f] | k\|fo h:ff] | slxn]sf+xL | la/n} | slxNo} klg |
| (= | d d]/f] jRrf;+u v]Ng dg k/fp5' . | ;w}e/L | w]/} h;f] | k\|fo h:ff] | slxn]sf+xL | la/n} | slxNo} klg |
| * !)= | d]/f] jRrf c;fWo} w]/} ?G5 . | ;w}e/L | w]/} h;f] | k\|fo h:ff] | slxn]sf+xL | la/n} | slxNo} klg |
| * !!= | d cfdf ePkl5 jGwgdf km;]sf] dx;'; u/]sf] 5' . | ;w}e/L | w]/} h;f] | k\|fo h:ff] | slxn]sf+xL | la/n} | slxNo} klg |
| * !@= | dnfO{ d]/f] aRrf;Fu l/; p7\5 . | ;w}e/L | w]/} h;f] | k\|fo h:ff] | slxn]sf+xL | la/n} | slxNo} klg |
| !#= | dnfO{ d]/f] jRrf ;+;f/sf] ;j}eGbf ;'Gb/ jRrf xf] eGg] nfU5 . | ;w}e/L | w]/} h;f] | k\|fo h:ff] | slxn]sf+xL | la/n} | slxNo} klg |
| * !$= | d]/f] aRrfn] dnfO{ lrlGtt agfp5 . | ;w}e/L | w]/} h;f] | k\|fo h:ff] | slxn]sf+xL | la/n} | slxNo} klg |
| * !%= | dnfO{ d]/f] jRrf;+u 8/ nfU5 . | ;w}e/L | w]/} h;f] | k\|fo h:ff] | slxn]sf+xL | la/n} | slxNo} klg |
| * !^= | dnfO{ d]/f] jRrfn] lbSs kf5{ . | ;w}e/L | w]/} h;f] | k\|fo h:ff] | slxn]sf+xL | la/n} | slxNo} klg |

**:sf]l/ª k|0ffnL :** ;w}e/LM ‘)’, w]/} h;f]M ‘!’, k|fo h:ff]M ‘@’ slxn]sf+xLM ‘#’, la/n}M ‘$‘’, slxNo} klgM ‘%’

Gff]6M * lrGx ePsf jfSof+zx?df pN6f] :sf]l/ª k|0ffnLsf] k|of]u ug'{xf];\ .

Note: *items need reverse scoring. Please do not make any changes on the Nepali Version of the Postpartum Bonding Questionnaire without the permission of the Corresponding Author.
