# Supplementary material for: Translation, validation, and factor structure of the Nepali version of postpartum bonding questionnaires (PBQ-N) among postpartum women in Nepal
Source: PLOS Glob Public Health. 2024 Jul 12;4(7):e0003469. doi: 10.1371/journal.pgph.0003469 (PMC11244797; doi:10.1371/journal.pgph.0003469)
Supplement: S2 Text — (DOCX) [file pgph.0003469.s004.docx]

**Postpartum Bonding Questionnaire- 16 Items_English Version**

**Directions:** Please indicate how often the following are true for you. There are no 'right' or 'wrong' answers. Select the answer which seems right in your recent experience.

|  |  |  | | | | | |
| --- | --- | --- | --- | --- | --- | --- | --- |
|  | I feel close to my baby | Always | Very often | Quite often | Sometimes | Rarely | Never |
| 1. * | I wish the old days when I had no baby would come back | Always | Very often | Quite often | Sometimes | Rarely | Never |
|  | I love to cuddle my baby  . | Always | Very often | Quite often | Sometimes | Rarely | Never |
| 1. * | I regret having this baby | Always | Very often | Quite often | Sometimes | Rarely | Never |
| 1. * | My baby winds me up. | Always | Very often | Quite often | Sometimes | Rarely | Never |
|  | I love my baby to bits | Always | Very often | Quite often | Sometimes | Rarely | Never |
|  | I feel happy when my baby smiles or laughs | Always | Very often | Quite often | Sometimes | Rarely | Never |
| 1. * | My baby irritates me | Always | Very often | Quite often | Sometimes | Rarely | Never |
|  | I enjoy playing with my baby | Always | Very often | Quite often | Sometimes | Rarely | Never |
| 1. * | My baby cries too much | Always | Very often | Quite often | Sometimes | Rarely | Never |
| 1. * | I feel trapped as a mother | Always | Very often | Quite often | Sometimes | Rarely | Never |
| 1. * | I feel angry with my baby | Always | Very often | Quite often | Sometimes | Rarely | Never |
|  | I think my baby is the most beautiful baby in the world | Always | Very often | Quite often | Sometimes | Rarely | Never |
| 1. * | My baby makes me feel anxious | Always | Very often | Quite often | Sometimes | Rarely | Never |
| 1. * | I am afraid of my baby | Always | Very often | Quite often | Sometimes | Rarely | Never |
| 1. * | My baby annoys me | Always | Very often | Quite often | Sometimes | Rarely | Never |

***Note*:** Scoring Guide (Always = 0, Very often = 1, Quite often =2, Sometimes = 3, Rarely = 4, Never = 5) with *items need reverse scoring. Factor Analysis results support these 16-item measure of the Postpartum Bonding Questionnaire to use among Nepalese Postpartum Mothers.
